# Crosstalk between *SOX* Genes and Long Non-Coding RNAs in Glioblastoma

**DOI:** 10.3390/ijms24076392

**Published:** 2023-03-28

**Authors:** Milena Stevanovic, Natasa Kovacevic-Grujicic, Isidora Petrovic, Danijela Drakulic, Milena Milivojevic, Marija Mojsin

**Affiliations:** 1 Laboratory for Human Molecular Genetics, Institute of Molecular Genetics and Genetic Engineering, University of Belgrade, 11042 Belgrade, Serbia; 2 Faculty of Biology, University of Belgrade, 11000 Belgrade, Serbia; 3 Serbian Academy of Sciences and Arts, 11000 Belgrade, Serbia

**Keywords:** *SOX* genes, lncRNA, cancer, GBM, GSC

## Abstract

Glioblastoma (GBM) continues to be the most devastating primary brain malignancy. Despite significant advancements in understanding basic GBM biology and enormous efforts in developing new therapeutic approaches, the prognosis for most GBM patients remains poor with a median survival time of 15 months. Recently, the interplay between the *SOX* (SRY-related HMG-box) genes and lncRNAs (long non-coding RNAs) has become the focus of GBM research. Both classes of molecules have an aberrant expression in GBM and play essential roles in tumor initiation, progression, therapy resistance, and recurrence. In GBM, *SOX* and lncRNAs crosstalk through numerous functional axes, some of which are part of the complex transcriptional and epigenetic regulatory mechanisms. This review provides a systematic summary of current literature data on the complex interplay between *SOX* genes and lncRNAs and represents an effort to underscore the effects of SOX/lncRNA crosstalk on the malignant properties of GBM cells. Furthermore, we highlight the significance of this crosstalk in searching for new biomarkers and therapeutic approaches in GBM treatment.

## 1. Introduction 

Grade IV glioma tumor, glioblastoma (GBM), is denoted as the most aggressive malignant brain tumor [1]. Regardless of surgical resections, radio- and chemotherapy, patients have a poor prognosis with an overall survival of less than two years [2]. Understanding the molecular mechanisms underlying the GBM is essential for the discovery of new, more efficient therapeutic approaches for this type of brain tumor. *SOX* (Sry-related HMG box) genes and lncRNAs (long non-coding RNAs) have specific expression profiles in GBM and their dysregulation is correlated with tumor promotion or suppression [3,4]. *SOX*/lncRNA crosstalk plays key roles in GBM progression and recurrence through numerous functional axes [5,6,7]. 

## 2. The Role of *SOX* Genes in Glioblastoma

**Figure 1:**
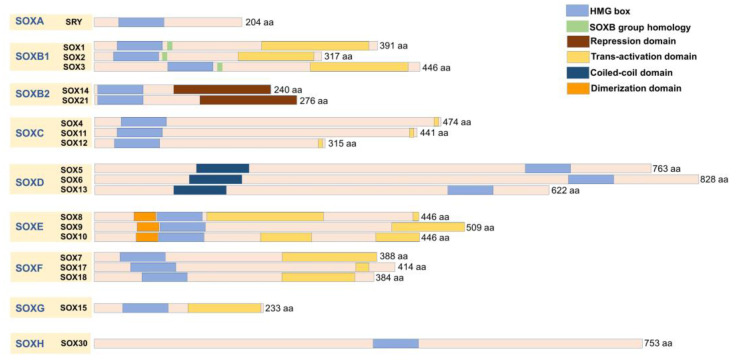
Schematic representation of grouping and structures of SOX proteins. Proteins are arranged in groups according to the HMG box domain homology (in sequence and position) and presented together with key structural features and functional domains. This scheme is based on the publications listed in the text.

*SOX* genes constitute a large family of diverse and well-conserved genes, comprising at least 20 family members in mammals that encode transcription factors (TFs) [8]. An HMG box of *Sox* genes encodes a domain that has an at least 50% amino acid sequence identity with that of *SRY* (sex-determining region Y) [9]. According to homology within the HMG domain and other structural motifs, this family is divided into eight distinct groups, designated from A to H [10]. Within particular groups (SOXB, SOXC, SOXD, and SOXE), the amino acid sequence identity of the HMG box domain remains >90%, although it decreases to ~60% between the distant groups [11]. SOX proteins within the same group also share homology outside the HMG box domain with regard to the amino acid sequence and the overall organization of protein domains (Figure 1) [10,12,13,14]. SOX TFs have indispensable roles during development, including maintenance of stem cell pluripotency, cell proliferation, cell fate decisions, germ layer formation, and the terminal differentiation of cells into tissues and organs [15]. Moreover, their roles are not restricted to development and involve the regulation of cell survival, regeneration, and homeostasis in adult tissues [16,17]. Like many other genes involved in the regulation of development, *SOX* genes are frequently dysregulated in cancer. A large amount of RNA-seq data revealed that *SOX* genes are aberrantly expressed in a variety of solid tumors, including bladder carcinoma [18], prostate carcinoma [19], renal cell carcinoma [20], liver carcinoma [21,22], sarcoma [22], cervical carcinoma [23], breast carcinoma [24], and lung carcinoma [25]. 

The roles of various SOX TFs have been described in the development and maintenance of brain tumors and GBM in particular [3]. SOX TFs regulate key processes related to tumor biology, including cell proliferation, migration, epithelial-to-mesenchymal transition (EMT), angiogenesis, apoptosis, and maintenance of the stemness of GSCs (glioma stem cells). In Table 1 we summarize their involvement in GBM pathophysiology by presenting them according to the groups in which they are classified.

The *SRY* gene, the only member of the *SOXA* group, is located on the Y chromosome. It is noticed that GBM is somewhat more frequent among males, with a male-to-female ratio of 1.6:1 [26], with females having a better overall survival than men [27]. However, no significant sex-related differences in transcriptomes of gliomas have been observed [28], but it seems that the existing differences in survival are attributed to the role of the male sex chromosome in patients’ samples. It has been shown that deletion of the *SRY* gene, loss of genes located on the Y chromosome, and complete loss of the Y chromosome in GBM samples negatively influence the survival of male patients [29]. SRY/chromosome Y status might partially explain the mechanisms underlying the observed sex disparities regarding incidence, prognosis, drug toxicity, clinical outcome, and therapeutic response in GBM [30]. However, the exact role of the *SRY* gene in GBM patients’ survival is not yet elucidated and needs further functional studies.

The role of the *SOXB1* subgroup (comprising *SOX1*, *SOX2*, and *SOX3*), and its member *SOX2* in particular, was extensively analyzed in GBM. *SOX1* is overexpressed in this malignancy, and a high level of expression correlates with shorter overall survival [31]. Additionally, *SOX1* expression is increased in GSCs and moderately promotes their self-renewal and proliferation [31]. *SOX2* is a marker of undifferentiated, proliferating cells and its expression is detected in all types of gliomas, in glioma cell lines and tumor-associated glial host cells [32,33,34,35]. High level of *SOX2* expression in GBM has been associated with tumor aggressiveness and worse prognosis [36]. Using SOX2 ChIP-seq and microarray analysis, Fang et al. found 4883 binding sites for SOX2 in the GBM cancer genome compared to IgG control ChIP-seq data, and identified 489 genes and 105 precursor microRNAs whose expression was altered in response to *SOX2* knockdown in GBM cells [37]. Among identified target genes, there were members of the *SOX* genes family, but also tumor suppressor genes, interleukins, and their receptors, previously described to play roles in GBM pathology. In vitro experiments showed that *SOX2* downregulation significantly decreases the migratory and invasive properties of GBM cells, indicating that SOX2 may serve as a potential therapeutic target in GBM [38]. Furthermore, it has been reported that SOX2 could contribute to the self-renewal and proliferation of glioma-initiating cells, which are important for the initiation, propagation, and recurrence of glioma [39]. Pan et al. performed wide bioinformatics analysis using ONCOMINE, GEPIA (Gene Expression Profiling Interactive Analysis), LinkedOmics, and CCLE (Cancer Cell Line Encyclopaedia) databases to assess the expression profiles and prognostic values of SOXB1 members in GBM [38]. Their analysis revealed that all three *SOXB1* members were upregulated in GBM to varying degrees, compared to the normal tissues, identifying only *SOX3* as a potential prognostic biomarker whose increased expression correlated with better overall survival [38]. In contrast, Lu et al. revealed a significantly higher expression level of *SOX3* in glioma compared with the normal tissues and correlated its overexpression with poor outcomes [40]. Our previous study revealed a higher level of *SOX3* expression in a subset of primary GBM samples compared to non-tumoral brain tissues and in a patient-derived GSC culture, suggesting that *SOX3* is required to maintain GSCs in an undifferentiated state [41]. However, we found that a high *SOX3* expression was not associated with the overall survival of GBM patients [41]. 

The *SOXB2* subgroup, consisting of *SOX14* and *SOX21*, which are closely related to *SOXB1* members, takes part in neurogenesis by counteracting the activities of SOXB1 proteins [42]. SOX2 and SOX21 target the same genes but with opposite effects since SOX2 contains an activating domain and SOX21 contains a repressing domain [43,44]. Targeted genes are regulated positively or negatively depending on the balance between these two TFs. An increase of SOX21 in glioma cells reduced tumor size and inhibited glioma progression in vivo by forming complexes with SOX2 protein, therefore changing the balance between these proteins in the tumor [45]. 

The literature data regarding the activity of *SOXC* group members (*SOX4* and *SOX11*) in GBM are contradictory. Zhang et al. reported that high *SOX4* expression was significantly associated with good prognosis and that SOX4 inhibited the growth of GBM cell lines [46]. In contrast, another group showed that SOX4 can drive glioma progression [47]. In two independent studies, *SOX11* was marked as a favorable prognostic factor in GBM, and its downregulation is associated with a considerable decrease in survival [48,49]. Hide et al. reported that glioma-initiating cells lost *SOX11* expression and that its overexpression prevented tumorigenesis by inducing the neuronal differentiation of these cells [48]. Korkolopoulou et al. showed that *SOX11* overexpression was correlated with improved overall survival in GBM, presenting *SOX11* tissue expression as an independent marker of favorable outcome, supporting its tumor suppressor function in astroglial tumors [49]. On the other hand, Weigle et al. revealed overexpression of *SOX11* in malignant glioma samples ranging from 5- to 600-fold [50], suggesting that *SOX11* expression reactivates during tumorigenesis in malignant gliomas. Accordingly, more comprehensive analyses are needed to delineate the exact roles of SOXC group members in GBM.

The *SOXD* group of potential tumor suppressors in GBM comprises *SOX5*, *SOX6*, and *SOX13*. The overexpression of *SOX5* in human glioma cells led to a reduction in clone formation and an inhibition of proliferation [51]. SOX5 can suppress PDGFB (platelet-derived growth factor B)-induced glioma development in mice by inhibiting cell proliferation and inducing acute cellular senescence through the regulation of p27Kip1 (cyclin-dependent kinase inhibitor 1B) and AKT1 (AKT serine-threonine protein kinase) [51]. It was shown that *SOX5* is a target of oncogenic miR-16 and miR-21 in GBM cells, which act as suppressors of *SOX5* expression [52]. The expression of *SOX6* was downregulated in GBM and a similar expression profile was described for *SOX13* [53]. On the other hand, data from the Human Protein Atlas database showed that the SOX6 protein level was higher in GBM cancer tissue compared to normal tissue [54]. Thus, *SOXD* group members might be considered significant favorable prognostic indicators.

The expression of *SOXE* group members (*SOX8*, *SOX9*, and *SOX10*) tends to be dysregulated in GBM compared to normal brain tissue [53]. More recent data indicate that *SOX9* expression in glioma tissues was significantly higher compared to corresponding non-neoplastic brain tissues and associated with poor clinical outcomes of patients [55]. Wang et al. have shown that the knockdown of *SOX9* in GBM cell lines markedly suppressed glioma cells’ sphere formation and reduced the expression of stem cell markers, indicating that SOX9 could be essential for GSC self-renewal [56]. Furthermore, serum levels of SOX9 and lncRNA-ANRIL (Antisense Non-coding RNA in the INK4 Locus) were higher in patients with glioma than in healthy people and were strongly associated with unfavorable prognosis [57]. A group of authors performed a comprehensive analysis of DNA methylation and gene expression profiles obtained from the GBM cohort and identified frequent tumor-specific methylation changes, including hypermethylation of the *SOX10* promoter [58]. This hypermethylation was associated with shorter survival in the analyzed cohort. According to current data, the upregulation of *SOX9* and downregulation of *SOX10* are correlated with poor clinical outcomes in GBM. 

Among the *SOXF* group members (*SOX7*, *SOX17*, and *SOX18*), *SOX7* expression is downregulated in GBM tissue samples and GBM cell lines [59,60]. Knockdown of *SOX7* promotes the proliferation of GBM cells, indicating that SOX7 may act as a tumor suppressor [59]. The exact roles of SOX17 and SOX18 are yet to be explored. A study on the epigenetic silencing of *SOX17* indicates that this gene was methylated in around one-fifth (20.31%) of patients with gliomas, and authors speculate that its epigenetic silencing may contribute to the upregulation of the Wnt signaling pathway or deregulation of the cell cycle in these tumors [61].

**Table 1:** Classification of *SOX* genes, their expression pattern in GBM, and correlation with the clinical outcome.

*SOX* Group	Members	Expression in GMB	Clinical Outcome	References
*SOXA*	*SRY*	Differentially expressed in GBM tissue samples	Downregulation was correlated with worse overall survival	[29]
*SOXB1*	*SOX1*	Overexpressed in GMB	Overexpression was correlated with poor prognosis	[31]
	*SOX2*	Overexpressed in GMB, a marker of undifferentiated proliferating cells of GBM	Overexpression was correlated with poor prognosis	[32,36]
	*SOX3*	Elevated expression in GBM	Overexpression correlated with better overall survival; or with poor outcome; or no effect on survival	[38,40,41]
*SOXB2*	*SOX14*	No data	No data	
	*SOX21*	Co-expressed with SOX2 in GBM, potential tumor suppressor	Elevated expression associated with low-risk score and better overall survival	[43]
*SOXC*	*SOX4*	Overexpressed in primary GBM tissues	High expression was correlated with a good prognosis	[46]
	*SOX11*	Overexpressed in malignant gliomas	High expression marked as a significant favorable prognostic indicator	[49]
	*SOX12*	No data	No data	
*SOXD*	*SOX5*	Low expression in GBM, suggested to act as a tumor suppressor	One study corelated its overexpression with poor prognosis	[51]
	*SOX6*	Downregulated or overexpressed	High expression correlated with lower survival rate	[53]
	*SOX13*	Downregulated in the majority of GBM samples	No data	[53]
*SOXE*	*SOX8*	Downregulated, could serve for predicting the differentiation status of glioma subtypes	No data	[53]
	*SOX9*	High expression in GBM	High expression was correlated with poor clinical outcome	[55]
	*SOX10*	Downregulated in GBM	Low expression was correlated with shorter survival rate	[58]
*SOXF*	*SOX7*	Downregulated in GBM tissue samples and cell lines	No data	[59]
	*SOX17*	No data	No data	
	*SOX18*	No data	No data	
*SOXG*	*SOX15*	Downregulated in GBM	Low expression was correlated with shorter survival	[62]
*SOXH*	*SOX30*	No data	No data	

The expression of *SOX15*, a *SOXG* group member, is decreased in GBM compared to normal tissue, and patients with a low expression of *SOX15* had shorter survival than those with high expression [62].

As presented, many *SOX* genes influence the malignant behavior of GBM cells. Their expression profile in GBM and correlation with the clinical outcome are summarized in Table 1. Many SOX members have already been recognized as promising candidates in the search for new therapeutic targets. The mechanisms behind SOX TFs’ activity in the induction and maintenance of malignant phenotype in GBM, and how their activity is controlled, including lncRNAs/miRNA/SOX axes, represent an interesting and insufficiently investigated area that deserves further research.

## 3. LncRNAs Acting through *SOX* Genes in Glioblastoma

LncRNAs are the type of RNA molecules longer than 200 nucleotides that do not encode proteins [63,64]. Most lncRNAs are transcribed from promoters by RNA polymerase II and possess a 5′ cap and poly(A) tail at a 3′ end [63,64]. LncRNAs can be divided into several groups according to their location in the genome and transcription relative to nearby protein-coding genes [65,66]. Sense lncRNAs are transcribed from the sense strand of corresponding protein-coding genes. Antisense lncRNAs are transcribed in the opposite direction to the protein-coding gene. Intronic lncRNAs are located and transcribed from introns of the protein-coding genes, while intergenic lncRNAs are located between protein-coding genes. Bidirectional lncRNAs are transcribed in an opposite direction to the protein-coding genes and their transcription starts 1000 base pairs away from the promoter region of protein-coding genes, whereas enhancer lncRNAs are transcribed from enhancer regions [65,66].

**Figure 2:**
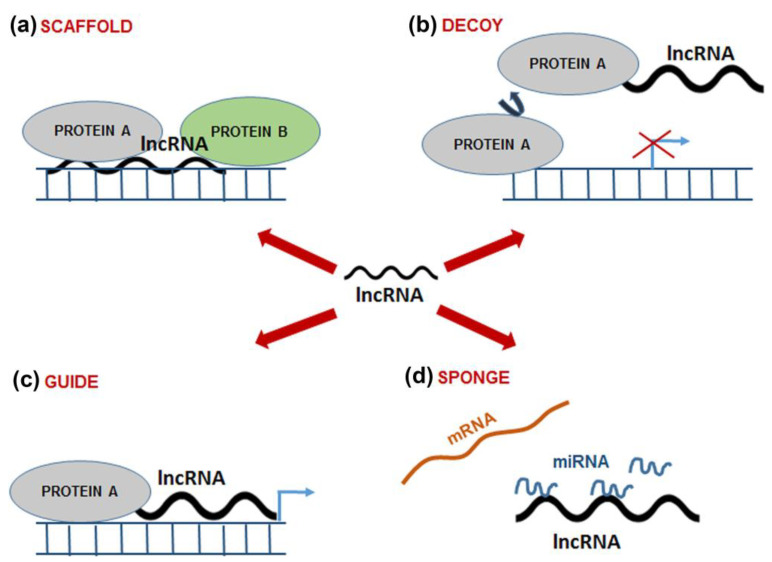
Functions of lncRNAs. LncRNAs can act as (**a**) scaffolds to assemble distinct proteins into complexes, (**b**) decoys to bind and sequester TFs and other regulatory proteins, (**c**) guides to recruit proteins, directing them to chromatin targets, (**d**) sponges that bind to miRNAs, preventing their interactions with mRNAs, leading to the repression as indicated by red cross mark. This summary is based on the publications listed in the text. LncRNA- Long non-coding RNA, miRNA-microRNA.

Based on their function, lncRNAs can be classified as scaffolds, decoys, guides, and sponges [67,68] (Figure 2). Scaffold lncRNAs assemble distinct proteins in a complex to activate or repress the expression of target genes (Figure 2a). Decoy lncRNAs bind and sequester TFs and other regulatory proteins, thus regulating their activity or interaction with the targets (Figure 2b). Guide lncRNAs recruit ribonucleoproteins and direct them to chromatin targets, causing changes in the expression of neighboring genes or genes located far away (Figure 2c). Some lncRNAs regulate mRNAs by competing with them for binding to shared miRNAs, acting as sponges that sequester miRNAs in the cells (Figure 2d) (reviewed in [67,68]).

LncRNAs have been recognized as important players in the cellular processes essential for normal function and in disease pathogenesis [69,70,71,72]. They are involved in the regulation of transcriptional and epigenetic regulatory mechanisms and the control of subcellular localization of their targets [69,70,71,72]. Aberrant expression of lncRNAs affects a range of cancer hallmarks and influences therapy efficiency and tumor recurrence [70,71,72]. 

The dysregulation of several lncRNAs has been detected in GBM cells, leading to the abnormal regulation of cancer-associated pathways functioning via targeting various miRNAs or genes/proteins and regulating various processes such as proliferation, invasion, migration, apoptosis, and metastasis, acting as both regulators and inhibitors [73]. Recently, emerging evidence has pointed to the interplay between *SOX* genes and lncRNAs in GBM.

**Figure 3:**
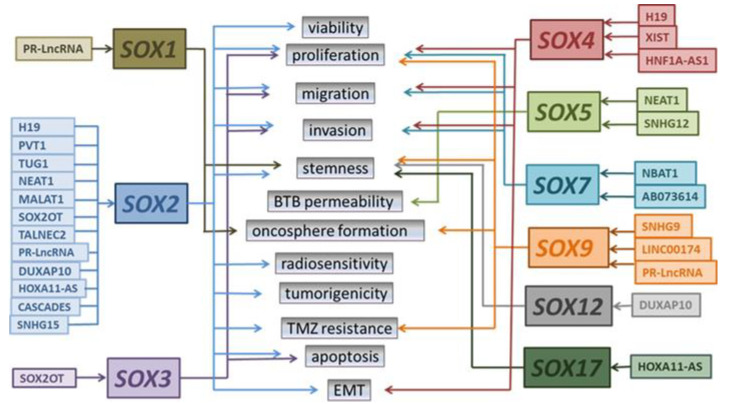
Specific lncRNAs operating through the modulations of *SOX* expression and key features of GBM cells and GSCs affected by lncRNAs. This summary is based on the previously reported publications listed in Table 2. BTB: Blood–tumor barrier; EMT: epithelial-to-mesenchymal transition. TMZ: temozolomide. MALAT1 (Metastasis Associated Lung Adenocarcinoma Transcript 1); NEAT1 (nuclear paraspeckle assembly transcript 1); TUG1 (taurine-upregulated gene 1); TALNEC2 (tumor-associated long non-coding RNA expressed on chromosome 2); PVT1 (plasmacytoma variant translocation 1); PR-LncRNA (p53-regulated LncRNAs); HOXA11-AS (Homeobox A11 antisense RNA); H19 (lncRNA encoded by the H19 gene); CASCADES (cancer stem cell associated distal enhancer of *SOX2*); XIST (X-inactive specific transcript); HNF1A-AS1 (HNF1A Antisense RNA 1); SNHG9 (small nucleolar RNA host gene 9); SNHG12 (small nucleolar RNA host gene 12); SNHG15 (small nucleolar RNA host gene 15); NBAT1 (neuroblastoma associated transcript 1); AB073614 (lncRNA AB073614); LINC00174 (Long Intergenic Non-Protein Coding RNA 174); DUXAP10 (Double Homeobox A Pseudogene 10); SOX2OT (SOX2 Over-lapping Transcript).

The roles of lncRNAs acting through SOX TFs and their revealed mechanisms of action are diverse. Detailed information about specific lncRNAs, their mechanism of action, *SOX* targets, and GBM and GSCs’ properties affected by them are presented in Table 2. A schematic representation of lncRNAs acting through *SOX* and their effects on the basic cellular processes in GBM are presented in Figure 3.

### 3.1. LncRNAs Acting through SOX with Tumor Suppressor Roles in GBM

Only two of the presented lncRNAs, PR-LncRNA (p53-regulated LncRNAs) and NBAT1 (neuroblastoma-associated transcript 1), have been confirmed to play tumor suppressor roles in glioma (Table 2, Figure 3). PR-LncRNAs act as negative regulators of cell survival and proliferation and contribute to p53 pro-apoptotic functions in colorectal cancer and glioma [74,94] (Table 2). Torres-Bayona et al. showed a gradual decrease in PR-LncRNA expression with advancing glioma grade, with the lowest expression in GBM samples [74]. Functional analyses also revealed a strong inverse correlation between the expression of PR-LncRNAs and SOX family members (SOX1, SOX2, and SOX9) in glioma clinical biopsies and glioma cells [74]. The authors suggested that SOX proteins are critical mediators of the PR-LncRNA activity in glioma and that PR-LncRNAs act upstream of SOX to regulate glioma cells’ activity [74]. 

NBAT1, another tumor suppressor lncRNA, exerts its activity through the regulation of SOX7 expression [75]. NBAT1 is downregulated in glioma tissues compared with that in the paracarcinoma tissues and its expression was decreased in patients with metastatic glioma compared with the controls. In addition, NBAT1 expression was significantly decreased in aggressive (grade III or IV) compared with low-grade (I or II) glioma. The downregulation of NBAT1 correlated with the upregulation of its target miR-21 and the downregulation of SOX7, a downstream target of miR-21 [75]. The formed NBAT1/miR-21/SOX7 axis represents the underlying molecular mechanism of NBAT1 functions in glioma [75].

### 3.2. LncRNAs Acting through SOX with Oncogenic Roles in GBM

The remaining lncRNAs presented in Table 2 (MALAT1, NEAT1, TUG1, TALNEC2, PVT1, H19, XIST, HNF1A-AS1, LINC00174, SNHG9, and SNHG15) are oncogenic and most of them bind particular miRNAs, preventing their interactions with specific *SOX* targets, thus functioning as competing endogenous RNAs (ceRNAs) (Table 2). The most common target of these lncRNAs is *SOX2* and consequently, their activity affects cellular processes regulated by SOX2 in GSCs and GBM cells such as stemness, viability, proliferation, migration, invasion, and chemo- and radioresistance (Table 2, Figure 3). LncRNAs specifically target cellular processes essential for maintaining the malignant phenotype in GBM through interplay with other *SOX* targets as well (Table 2, Figure 3). For example, H19/miR-130a-3p/SOX4 and XIST/miR-133a/SOX4 axes facilitate EMT by increasing the SOX4 transcriptional activity and its effects on the TGF-β/Smad pathway and Wnt signaling [84,86].

The other regulatory mechanisms are also involved in *SOX*/lncRNA interplay in GBM (Table 2, Figure 3). LncRNA CASCADES is a *SOX2* super-enhancer-associated lncRNA [85]. Super-enhancers represent clusters of enhancers in close genomic proximity that can work as independent regulatory regions or as a part of a large transcription regulatory network to enable the gradient expression of genes they regulated [95,96]. They regulate cell identity genes and are considered to be essential for the maintenance of the oncogenic potential of cancer cells [97]. Super-enhancer-associated lncRNAs, lncRNAs transcribed from super-enhancers, have emerged as master regulators of cell fate determination and differentiation since they regulate the expression of genes essential for these processes by transcription factor trapping, chromatin modifications, recruitment of the RNA Pol II complex, and removal of the co-repressor function [98,99]. Shahzad et al. discovered lncRNA CASCADES transcribed from a distal super-enhancer of *SOX2* [85]. They revealed that CASCADES is an epigenetic regulator of *SOX2* in GSCs and an essential factor for the maintenance of stemness in these cells [85]. High CASCADES expression was detected in both primary and recurrent IDH-WT gliomas and correlated with poor overall survival [85]. The authors proposed a model in which CASCADES acts as a “transcription factor-trapper” to facilitate the expression of the *SOX2* gene [85]. They found that YY1 (Yin Yang 1), a transcription factor that mediates enhancer-promoter structural interactions, binds to the CASCADES enhancer and proximal promoter of the *SOX2* gene. They suggest that the binding of Rad21 (Double-strand-break repair protein rad21 homolog) to the CASCADES enhancer and proximal promoter of *SOX2* implicates chromatin looping, RNA Pol II binding to both elements, and the simultaneous transcription of CASCADES and *SOX2* gene. LncRNA CASCADES then modulates the activity of *SOX2* super-enhancer in a positive feedback loop by entrapping YY1 at the proximal promoter of the *SOX2* gene, facilitating its transcription [85]. Since the authors showed that the knockdown of CASCADES promotes the neuronal differentiation of GSCs, CASCADES represents a promising therapeutic target for the potential differentiation therapy of GBM [85].

LncRNA DUXAP10 (Double Homeobox A Pseudogene 10) binds directly to RNA-binding protein HuR (human antigen R) in the cytoplasm and suppresses its translocation to the nucleus [92]. In the cytoplasm, HuR directly binds to *SOX12* mRNA and enhances its stability, thus increasing *SOX12* expression [92]. By this mechanism, DUXAP10 promotes the stemness of GBM cells. It is interesting to point out that the HuR/Sox12 axis increases the expression of *SOX2* and that, based on in silico predictions, HuR can also bind *SOX1*, *SOX10*, *SOX11*, and *SOX13* [92].

Some of the *SOX*/lncRNA interactions are part of the complex regulatory mechanisms involved in the malignant behavior of glioma cells. SNHG12 was upregulated in glioma and its expression was positively correlated with the glioma grades [89]. Liu et al. profiled the expression of TDP43 (TAR-DNA binding protein 43), SNHG12, miR-195, SOX5, and Gelsolin in GBM and revealed that their complex interplay drives glioma malignant progression [89]. They showed that TDP43 exerts its oncogenic role in glioma by the direct binding and stabilization of lncRNA SNHG12 [89]. In addition, they showed that tumor suppressor miR-195, downregulated in gliomas, targeted SNHG12 in a sequence-specific manner and suggested the reciprocal repression feedback loop between SNHG12 and miR-195. The next cascade in this complex mechanism involved *SOX5*, upregulated in glioma, as a downstream target of miR-195. In turn, SOX5 upregulated the expression of oncogene Gelsolin and SNHG12, thus forming a positive feedback loop of SNHG12/miR-195/SOX5 [89]. Recently, SNHG12 has been recognized as a potential biomarker since its expression correlated with clinical characteristics and prognosis in various cancers, but also as a potential therapeutic target due to its involvement in the unfolded protein response-adaptive pro-survival mechanism exploited by many cancer cells [100,101].

SOX proteins also function as mediators of lncRNA interplay with signaling pathways whose activity is impaired in glioma. LncRNA AB073614 induces the activity of the Wnt/β-catenin signaling pathway by downregulating the SOX7 expression and promoting the progression of glioma [91]. Additionally, NOTCH1 activation in GSCs induces the expression of the lncRNA TUG1. TUG1 increases the expression of *SOX2* by sponging miR-145, thus promoting the stemness of GSCs [79].

Besides GSCs and GBM cell lines, SOX/lncRNA interplay also regulates the functions of glioma endothelial cells (GECs). LncRNA NEAT1 binds miR-181d-5p and upregulates the expression of *SOX5*, the downstream target of miR-181d-5p. SOX5 binds to the promoter regions of ZO-1 (Zonula Occludens-1), occluding, and claudin-5 and regulates the expression of tight junction proteins in GECs, thus controlling the permeability of the blood–tumor barrier (BTB), the limiting factor for drug delivery in glioma treatments [78].

The interplay of *SOX* genes and their overlapping transcripts and SOX antisense RNA will be described in detail in the next section.

## 4. SOX Overlapping Transcripts and SOX Antisense RNA

There are only a few papers describing SOX1 overlapping transcript (SOX1OT) (ENSG00000224243). It is lncRNA that maps to human chromosome 13 [102] and has a complex structure encompassing at least 2 potential transcription start sites, 10 exons, and 11 different transcript variants [103]. The *SOX1* gene is embedded within an intron of a SOX1OT, and their expressions correlate during neural differentiation as well as in cancer cell lines, such as teratocarcinoma (NTera) and breast carcinoma cell lines [103]. Additionally, it was demonstrated that the axis SOX1OT transcript variant 1/HDAC10/SOX1/ASCL1 has important functions in dorsal cortical and ventral GABAergic neuronal differentiation [104]. To the best of our knowledge, there are no data about SOX1OT expression in GBM. Having in mind that SOX1 has oncogenic activity in GBM and that *SOX1* and SOX1OT expressions correlate in some cancer cell lines, it would be interesting to analyze SOX1OT expression in GBM. 

The SOX2 overlapping transcript (SOX2OT) (ENST00000485035.1) is an evolutionarily conserved lncRNA mapping to human chromosomal locus 3q26.3 [105,106]. It consists of ten exons, has more than two transcription start sites, and produces at least eight transcript variants (reviewed in [107]), which demonstrate various expression profiles in diverse cell or tissue types [108]. SOX2OT is expressed in mouse embryonic stem cells and its expression is downregulated upon induction of embryoid body differentiation [105]. Furthermore, it was shown that during the neural differentiation of mouse embryonic stem cells, SOX2OT and *SOX2* RNA levels are inversely correlated [109]. In the developing mouse cerebral cortex, SOX2OT interacts with the epigenetic regulator YY1, represses neural progenitor proliferation, and promotes neuronal differentiation [110]. During central nervous system (CNS) development, expression of SOX2OT is upregulated [111], and the highest level of its expression was detected in the human brain and spinal cord [112,113]. 

**Figure 4:**
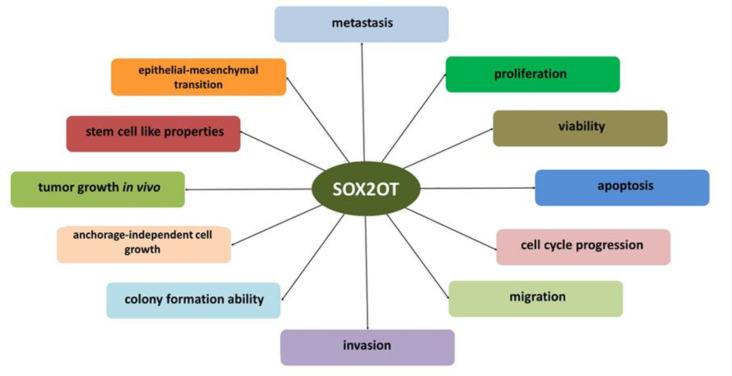
Tumor cells’ properties influenced by SOX2OT. This summary is based on the publications listed in Table 3.

An increased level of SOX2OT expression is observed in various tumors, and cell properties affected by SOX2OT in tumor cells in which SOX2OT operates through the modulations of *SOX* gene expression are summarized in Table 3 and Figure 4.

It was demonstrated that high SOX2OT expression is associated with the poor survival/prognosis of patients with lung cancer [122], gastric cancer [123,124], hepatocellular carcinoma [125,126], ovarian cancer [127], pancreatic ductal adenocarcinoma [6], cholangiocarcinoma [116], osteosarcoma [117], nasopharyngeal carcinoma [128], bladder cancer [119] and prostate cancer [129]. 

Among the four TFs (SOX2, IRF4, AR, and SOX3) able to bind directly to the SOX2OT promoter and stimulate its transcription, two are SOX TFs (reviewed in [111]). The *SOX2* gene, embedded in the intronic region of SOX2OT, is a target of SOX2OT, and both the *SOX2* gene and SOX2OT are transcribed in the same orientation [130]. Furthermore, SOX3 could directly bind to the SOX2OT promoter, forming a positive feedback loop (Table 2) [93]. 

The increased expression of SOX2OT was detected in glioma tissues, and its expression was positively correlated with the tumor grade [93]. GBM patients with higher SOX2OT expression levels had poor prognosis and higher risk of relapse compared to patients with lower SOX2OT levels [7,131]. Furthermore, the expression of SOX2OT is higher in human GBM cell lines and GSCs compared to human astrocytes; a higher level of SOX2OT was found in GSCs derived from U87 and U251 GBM cells compared to the expression detected in parental cell lines, respectively [93]. The literature data revealed that the SOX2OT/miR-192-5p/RAB2A axis and ERK pathway stimulate GBM cell growth [132]. Moreover, it has been demonstrated that a high level of SOX2OT expression can promote the proliferation, migration, and invasion of GSCs and inhibits apoptosis via the SOX2OT-miR-194-5p/miR-122-SOX3-TDGF-1 pathway [93]. SOX2OT knockdown in GBM cells leads to changes in the expression of genes linked to DNA replication, development, cell cycle regulation and neuronal differentiation [133]. Furthermore, the expression of SOX2OT is increased in recurrent GBM patient samples and TMZ-resistant U87 and U251 GBM cells [7]. Furthermore, it was demonstrated that SOX2OT promotes proliferation, inhibits apoptosis, and decreases TMZ sensitivity by upregulating *SOX2* expression, which activates the Wnt5a/β-catenin signaling pathway (Table 2) [7]. 

The literature data revealed that SOX2OT influences the malignant behavior of tumor cells via miRNAs. Namely, SOX2OT downregulates the expression of miRNA-942-5p in breast cancer cells, miR -142 and miR-22 in osteosarcoma cells, miR-200c in bladder cancer cells, miR-122 in hepatocellular carcinoma cells, miR-452-5p in prostate cancer cells, miR-146b-5p in nasopharyngeal carcinoma cells, and miR-144-3p in multiple myeloma cells [119,128,129,134,135,136,137]. The expression of these miRNAs is downregulated in gliomas as well [138,139,140,141,142,143,144,145]. Considering these data, it would be interesting to analyze if SOX2OT influences the malignant characteristics of GBM cells via these miRNAs.

Having in mind all these data, it may be concluded that SOX2OT represents a promising therapeutic target in different types of diseases, including GBM. One of the approaches to decrease the expression of SOX2OT in GBM cells might be to test different bioactive compounds, given that successful targeting of SOX2OT transcript variant 7 with EGCG, a polyphenol in green tea, has already been demonstrated in osteosarcoma cells. Namely, EGCG increased doxorubicin-induced inhibition of osteosarcoma cell growth through downregulation of the expression of SOX2OT transcript variant 7 [146]. 

Another level of complexity of the *SOX*/lncRNA interplay in GBM is attained by the activity of SOX antisense RNA [147]. LncRNA SOX21-AS1 (SOX21 antisense RNA 1) shares a bidirectional promoter with the *SOX21* gene, and their expression is simultaneously regulated in some cancers, such as oral carcinoma [147]. 

## 5. Potential of lncRNAs for Prognosis and Therapy in GBM

### 5.1. LncRNAs as Prognostic Markers in GBM

In recent years, different bioinformatics tools and databases containing molecular and clinical data on a vast number of cancer patients—The Cancer Genome Atlas (TCGA, https://cancergenome.nih.gov/tcga) database and the Chinese Glioma Genome Atlas (CGGA, http://www.cgga.org.cn, [148])—have proven to be extremely valuable in the search for panels of disease-specific, differentially expressed lncRNAs that could serve as novel diagnostic or prognostic tools in glioma, including GBM. Sets of lncRNAs related to the immune system [149,150], autophagy [151], EMT [152], pyroptosis [153], ferroptosis [154], and lncRNAs with methylated promoters [155] have been explored to develop reliable and biologically relevant lncRNA signatures for predicting survival of patients with GBM. To acquire better insights into the biological function of lncRNA signatures and molecular mechanisms/pathways through which they exert their function in GBM, a growing amount of research is focused on in silico functional analyses to establish lncRNA-miRNA-mRNA-associated ceRNA regulatory networks in GBM [156,157,158,159,160]. Peng et al. constructed a ceRNA co-expression network consisting of two lncRNAs (NORAD, XIST), five miRNAs (miR-3613, miR-371, miR-373, miR-32, miR-92), and two mRNAs (LYZ, PIK3AP1), which might serve as a prognostic biomarker in GBM [157]. Based on the enrichment analysis, the authors hypothesized that the identified ceRNA network influences immune activities and the tumor microenvironment [157]. Li et al. stressed the importance of considering the GBM subtypes when evaluating the co-expression of lncRNA–mRNA pairs in GBM [160]. They revealed that a substantial fraction of lncRNA–mRNA regulation relationships are subtype-specific, and they identified subtype-specific modules in which lncRNAs and mRNAs compete with each other for miRNAs [160]. Classic- and mesenchymal-specific modules were mostly related to biological functions such as cell proliferation, apoptosis, and migration, while proneural- and neural-specific modules were mainly implicated in DNA damage and cell cycle dysregulation [160]. Some of the modules showed the potential to be prognostic markers of patients with classic and mesenchymal subtypes of GBM [160]. The functional validation of lncRNAs within identified ceRNA regulatory networks will also uncover potential therapeutic targets in GBM. 

### 5.2. Approaches for Therapeutic Targeting of lncRNAs 

**Figure 5:**
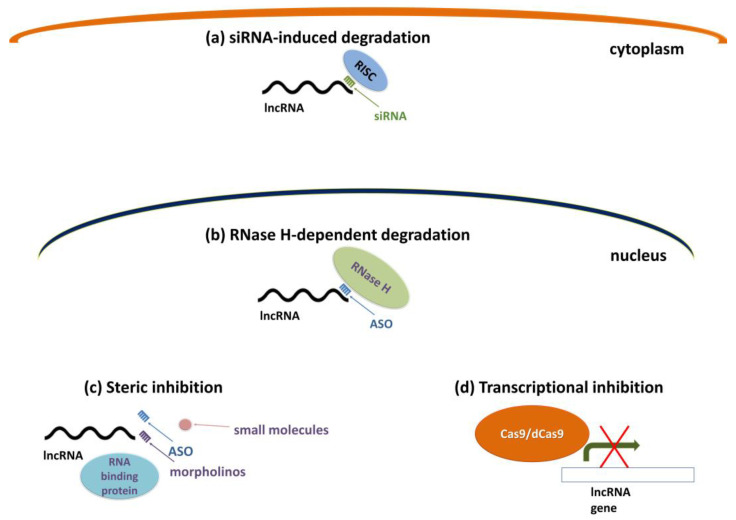
Approaches for targeting nuclear and cytosolic lncRNAs. (**a**) siRNAs can target cytosolic lncRNAs and stimulate degradation by recruiting the RISC complex; (**b**) ASOs can target and degrade nuclear lncRNAs via the RNase H-dependent mechanism; (**c**) uniformly modified ASOs, morpholinos, or small molecules can prevent lncRNA–protein interactions or the formation of secondary structure; (**d**) transcriptional inhibition by genome editing CRISPR/Cas9 methodology or dead-Cas9 fused to a repressor complex as indicated by red cross mark. References are included in the main text.

Based on lncRNAs’ diverse mechanisms of action and subcellular localization, lncRNAs in cancers can be targeted by several approaches (Figure 5) (reviewed in [161]): (a) degradation of cytosolic lncRNAs using small interfering RNAs (siRNAs), which includes the recruitment of the RISC complex (RNA-induced silencing complex), RNAse dicer and endonuclease Argonaut2-dependent pathway [162,163]; (b) RNAse H-dependent degradation of nuclear-located lncRNAs by chemically modified antisense oligonucleotides (ASOs); (c) steric inhibition of specific lncRNA–protein interactions or prevention of secondary structure formation using uniformly modified ASOs, morpholinos or small-molecule inhibitors; and (d) usage of CRISPR (clustered regularly interspaced short palindromic repeats)/Cas9 technology to knockout specific lncRNAs or CRISPR-mediated interference (CRISPRi), to repress the transcription of lncRNAs by recruiting catalytically inactive Cas9 enzyme, fused to a repressor complex, to the transcription start site using a single-guide RNA [164].

Several in vitro and in vivo studies using RNAi- or oligonucleotide-based strategies or CRISPR/Cas9 editing to target lncRNAs have been successful in the treatment of different cancers, including GBM. Kim et al. used anti-MALAT1 siRNA, encapsulated in a tumor-targeting and blood–brain barrier-crossing immunoliposome, to reduce the growth, migratory potential, and stemness of TMZ-resistant GBM cells, which was accompanied by their improved sensitivity to TMZ [165]. A combination of TMZ treatment with MALAT1 silencing inhibited tumor growth and increased survival in the orthotopic xenograft model of GBM [165]. Lentiviral delivery of shRNA targeting MALAT1 repressed proliferation and induced the cell cycle arrest and apoptosis of GBM cells in vitro [166]. Silencing of MALAT1 was also notably correlated with smaller tumor size and longer median survival time of xenograft transplanted mice [166]. The knockdown of MALAT1 by specific ASOs in breast and lung cancer mouse models caused slower tumor growth and a decrease in metastasis [167,168]. Silencing of NEAT1 by specific siRNA or chemically stabilized ASOs (locked nucleic acid (LNA)-GapmeRs) sensitized human breast carcinoma (MCF-7) and osteosarcoma (U2OS) cells to both chemotherapy and p53 reactivation therapy [169]. A dual sgRNA CRISPR/Cas9 system has been successfully used to knockout NEAT1, leading to GBM cell apoptosis and the inhibition of their proliferation, clone formation, and invasion [170]. In addition, tumor growth and invasion were hampered in an orthotopic mouse model after the CRISPR/Cas9-mediated depletion of NEAT1 [170]. The systemic delivery of ASO targeting TUG1, coupled with cRGD peptide-conjugated polymeric micelles that enable ASO accumulation within the tumor, induced GSC differentiation and potently reduced tumor growth in an intracranial xenograft mouse model [79].

To date, about a dozen RNA-based therapeutics have been approved by the Food and Drug Administration and/or the European Medicines Agency (reviewed in [171]). They comprise either siRNAs or chemically modified ASOs that target specific mRNAs, or ASOs that alter pre-mRNA splicing in the target organs such as muscle, liver, and CNS [172,173,174,175]. However, there have been no lncRNA-based therapeutics in clinical trials so far. On the other hand, lncRNAs have been investigated in clinical trials as potential biomarkers for the detection of gastric cancer (NCT05397548), in the diagnosis of hepatocellular carcinoma (NCT05088811) and lung cancer (NCT03830619), for the prediction of immunotherapy response of gastric cancer (NCT05334849), and a distinction between malignant and benign thyroid tumors (NCT04594720).

## 6. Future Directions

As presented in this paper, a vast amount of data revealed that *SOX*/lncRNA axes affect almost all features of GBM and GSCs and indicate that the majority of lncRNAs act through *SOX2*. Since the direct targeting of SOX2, as an “undruggable” TF [176], has little therapeutic value, targeting signal molecules upstream of SOX2 is a promising alternative approach. Another promising target is the NEAT1/miR-181d-5p/SOX5 axis that regulates the permeability of BTB in GECs, since the selective increase of BTB permeability and more efficient drug delivery are some of the ultimate challenges in the chemotherapeutic treatment of GBM. However, the clinical relevance of *SOX*/lncRNA axes and precise delineation of individual contributions of multiple lncRNAs targets is needed for a realistic assessment of their therapeutic potential.

Since lncRNAs exert their functions via transcriptional, post-transcriptional, and epigenetic mechanisms, large-scale integrated analyses are needed to understand in detail the underlying molecular mechanisms, before stepping into clinical trials. Research on *SOX*/lncRNA axes as potential therapeutic targets should be focused on comprehensive preclinical studies, with a special emphasis on the ability of potential therapeutics to cross the BBB. Studies combining therapies targeting *SOX*/lncRNA axes and conventional chemotherapy are also needed, keeping in mind that the administration of siRNA against MALAT1 sensitized GBM to TMZ [165]. Another level of complexity is the tumor’s recurrence. It has been revealed that both lncRNAs and SOX proteins show different expression profiles in primary and recurrent GBM tumors [177,178]. 

Important issues that need to be considered before the potential therapeutic exploitation of *SOX*/lncRNA interplay in GBM when using nucleic acid-based approaches include off-target effects, toxicity, innate immune response to foreign RNA, short half-life, and bioavailability. 

## 7. Concluding Remarks

In recent decades, a new world of regulatory ncRNAs has emerged, opening avenues for the development of the next generation of RNA therapeutics. Specificity, low toxicity, and the ability to act jointly with other regulatory molecules make them advantageous in targeting the complex pathways in pathological conditions.

Crosstalk between *SOX* genes and lncRNAs has a significant role at various stages of tumor onset and progression in GBM. The comprehensive assessment of *SOX*/lncRNA interplay might facilitate the identification of the molecular pathways underlying the pathogenesis of this type of cancer and establish novel therapeutic strategies for GBM treatment.

## Figures and Tables

**Table 2:** LncRNAs acting through *SOX* genes in GBM: MALAT1 (Metastasis Associated Lung Adenocarcinoma Transcript 1), NEAT1 (nuclear paraspeckle assembly transcript 1), TUG1 (taurine-upregulated gene 1), TALNEC2 (tumor-associated long non-coding RNA expressed on chromosome 2), PVT1 (plasmacytoma variant translocation 1), H19 (lncRNAs encoded by the H19 gene), XIST (X-inactive specific transcript), HNF1A-AS1 (HNF1A Antisense RNA 1), LINC00174 (Long Intergenic Non-Protein Coding RNA 174), SNHG9 (small nucleolar RNA host gene 9), and SNHG15 (small nucleolar RNA host gene 15).

LncRNA	*SOX* Targets	Mechanism of Action	Glioma Cells Properties Affected by Modulation of *SOX* Expression	Reference
lncRNA with tumor suppressor roles				
PR-LncRNA	*SOX1* *SOX2* *SOX9*	decreases expression of *SOX1*, *SOX2*, and *SOX9*	decreases the oncosphere formation ability and self-renewal potential of GSCs	[74]
NBAT1	*SOX7*	NBAT1/miR-21/*SOX7* axis	inhibits proliferation, migration, and invasion of GBM cells	[75]
lncRNA with oncogenic roles				
MALAT1	*SOX2*	MALAT1/miR-129/*SOX2* axis	enhances viability and proliferation of GSCs	[76]
NEAT1	*SOX2*	NEAT1/miR-132/*SOX2* axis	promotes viability, migration, and invasion of GBM cells	[77]
	*SOX5*	NEAT1/miR-181d-5p/*SOX5* axis that reduces expression of tight junction proteins ZO-1, occludin, and claudin-5	impaired BTB permeability in GECs	[78]
TUG1	*SOX2*	TUG1/miR-145/*SOX2* axis	maintains the stemness and tumorigenicity of GSCs	[79]
TALNEC2	*SOX2*	TALNEC2/miR-21/*SOX2* axis TALNEC2/miR-191/*SOX2* axis	promotes mesenchymal transformation and stemness and decreases radiosensitivity of GSCs	[80]
PVT1	*SOX2*	PVT1/miR-365/ELF4/*SOX2* axis	promotes proliferation, migration, invasion, and temozolomide (TMZ) resistance of GBM cells	[81]
HOXA11-AS	*SOX2* *SOX17*	increases expression of *SOX2* and *SOX17*	promotes stemness of GBM cells and glioma progression in vivo	[82]
H19	*SOX2*	increases expression of *SOX2*	promotes proliferation, migration, stemness, and TMZ resistance in GBM cells	[83]
	*SOX4*	H19/miR-130a-3p/*SOX4* axis	promotes migration, invasion, and neurosphere formation, and facilitates EMT	[84]
CASCADES	*SOX2*	*SOX2* super-enhancer associated lncRNA that modulates activity of *SOX2* in a positive feedback loop	promotes stemness of GSCs	[85]
XIST	*SOX4*	XIST/miR-133a/*SOX4* axis	promotes proliferation, invasion, migration, and EMT of GBM cells	[86]
HNF1A-AS1	*SOX4*	HNF1A-AS1/miR-32-5p/*SOX4* axis	promotes proliferation, migration, and invasion, and inhibits apoptosis of GBM cells	[87]
SNHG9	*SOX9*	SNHG9/miR-326/*SOX9* axis	promotes the growth of GSCs	[88]
SNHG12	*SOX5*	TDP43/SNHG12/miR-195/SOX5 axis that promotes expression of oncogene Gelsolin SOX5 activates SNHG12 forming a feedback loop	promotes proliferation, migration, and invasion and inhibits apoptosis of GBM cells	[89]
SNHG15	*SOX2*	SNHG15/miR-627-5p/*SOX2* axis	promotes GBM tumorigenesis, decreases sensitivity towards TMZ treatment	[90]
AB073614	*SOX7*	AB073614 induces Wnt/β-catenin signaling activity by downregulation of *SOX7* expression	promotes proliferation, migration, and invasion of GBM cells	[91]
LINC00174	*SOX9*	LINC00174/miR-138-5p/*SOX9* axis	promotes proliferation, cell cycle progression, and increases chemoresistance to TMZ in GBM cells	[5]
DUXAP10	*SOX12* *SOX2*	recruiting HuR to the cytoplasm enhancing *SOX12* mRNA stability increases expression of *SOX2*	promotes stemness of GBM cells facilitates growth of GSCs	[92]
SOX2OT	*SOX2*	SOX2OT binds RNA demethylase ALKBH5 which regulates *SOX2* expression via RNA demethylation	inhibits cell apoptosis, promotes cell proliferation, and TMZ resistance	[7]
		SOX2OT/*SOX2*/Wnt5a/β-catenin axis		[7]
		SOX2OT/miR-194-5p/*SOX3* axis		
	*SOX3*	SOX2OT/miR-122/*SOX3* axis	inhibits proliferation, migration, and invasion of GSCs, and promotes apoptosis of GSCs	[93]

**Table 3:** Signaling axes by which SOX2OT influences properties of tumor cells.

Tumor Cells	Signaling Axis	Cell Properties Affected by SOX2OT	Reference
breast cancer cells	SOX2OT/SOX2	Ectopic expression of SOX2OT reduces proliferation and increases breast cancer cell anchorage-independent growth	[114]
pancreatic ductal adenocarcinoma cells	SOX2OT/miR-200/SOX2	SOX2OT promotes EMT, stem cell-like properties, invasion and metastasis	[6]
	YY1/SOX2OT/SOX2	Ectopic expression of SOX2OT promotes cell proliferation and colony formation capacity	[115]
cholangiocarcinoma cells	IRF4/SOX2OT/SOX2/PI3K/AKT	SOX2OT overexpression increases cell proliferation, decreases apoptosis rate and enhances migratory and invasion abilities in vitro, and metastatic ability in vivo	[116]
osteosarcoma cells	SOX2OT/SOX2	SOX2OT overexpression promotes cell proliferation, increases colony formation ability, elevates migration, and invasion capabilities, and increases the expression of cancer stem cell biomarkers	[117]
cervical cancer cells	SOX2OT/SOX2	SOX2OT knockdown suppresses cell viability, arrests cell cycle and ameliorates migration and invasion abilities of cells	[118]
bladder cancer cells	SOX2OT/miR-200c/SOX2	SOX2OT knockdown inhibits the stemness phenotype (self-renewal, migration, invasion, and tumorigenicity) of bladder cancer stem cells	[119]
colorectal cancer cells	SOX2OT/miR-194-5p/SOX5	SOX2OT silencing suppresses cell proliferation, migration, and invasion in vitro, and inhibits tumorigenesis in vivo	[120]
esophageal squamous cell carcinoma cells	SOX2/SOX2OT	SOX2OT overexpression promotes cell growth	[121]

## Data Availability

Not applicable.
